# FemZone trial: a randomized phase II trial comparing neoadjuvant letrozole and zoledronic acid with letrozole in primary breast cancer patients

**DOI:** 10.1186/1471-2407-14-66

**Published:** 2014-02-05

**Authors:** Peter A Fasching, Sebastian M Jud, Maik Hauschild, Sherko Kümmel, Martin Schütte, Matthias Warm, Volker Hanf, Dieter Grab, Jutta Krocker, Elmar Stickeler, Rolf Kreienberg, Thomas Müller, Thorsten Kühn, Christopher Wolf, Steffen Kahlert, Stefan Paepke, Michael Berghorn, Mathias Muth, Monika Baier, Birgit Wackwitz, Rüdiger Schulz-Wendtland, Matthias W Beckmann, Michael P Lux

**Affiliations:** 1 University Breast Center, Department of Gynecology and Obstetrics, Erlangen University Hospital, Friedrich Alexander University of Erlangen–Nuremberg, Erlangen Comprehensive Cancer Center Erlangen-EMN, Erlangen, Germany; 2 Departement of Gynecology and Obstetrics, Spital Rheinfelden, Switzerland; 3 Breast Center, Klinikum Essen-Mitte, Essen, Germany; 4 Department of Gynecology and Obstetrics, Essen University Hospital, Essen, Germany; 5 Department of Gynecology and Obstetrics, Cologne University Hospital, Cologne, Germany; 6 Kliniken der Stadt Köln Holweide, Cologne, Germany; 7 Clinic for Gynecology and Obstetrics "Nathanstift" Klinikum Fürth, Fürth, Germany; 8 Department of Gynecology and Obstetrics, Klinikum Harlaching, Munich, Germany; 9 Department of Gynecology and Obstetrics, Sana-Klinikum Lichtenberg, Oskar-Ziethen-Krankenhaus, Berlin, Germany; 10 Department of Gynecology and Obstetrics, Freiburg University Hospital, Freiburg, Germany; 11 Department of Gynecology and Obstetrics, Ulm University Hospital, Ulm, Germany; 12 Frauenklinik, Klinikum Hanau, Hanau, Germany; 13 Department of Gynecology and Obstetrics, Klinikum Esslingen, Esslingen, Germany; 14 Medizinisches Zentrum Ulm, Ulm, Germany; 15 Frauenklinik und Poliklinik der Technischen Universität München, Munich, Germany; 16 Frauenklinik Grosshadern, Universitätsklinik der Ludwig-Maximilians-Universität, Munich, Germany; 17 Frauenklinik, Allgemeines Krankenhaus Celle, Celle, Germany; 18 Novartis Pharma GmbH, Nuremberg, Germany; 19 Institute of Diagnostic Radiology, Erlangen University Hospital, Friedrich Alexander University of Erlangen–Nuremberg, Erlangen, Germany

**Keywords:** Zoledronic acid, Neoadjuvant treatment, Breast cancer, Letrozole, Aromatase inhibitors, Bisphosphonates

## Abstract

**Background:**

The objective of this prospectively randomized phase II trial (Trial registration: EUCTR2004-004007-37-DE) was to compare the clinical response of primary breast cancer patients to neoadjuvant therapy with letrozole alone (LET) or letrozole and zoledronic acid (LET + ZOL).

**Methods:**

Patients were randomly assigned to receive either LET 2.5 mg/day (n = 79) or the combination of LET 2.5 mg/day and a total of seven infusions of ZOL 4 mg every 4 weeks (n = 89) for 6 months. Primary endpoint was clinical response rate as assessed by mammogram readings. The study was terminated prematurely due to insufficient recruitment. We report here on an exploratory analysis of this data.

**Results:**

Central assessment of tumor sizes during the treatment period was available for 131 patients (66 LET, 65 LET + ZOL). Clinical responses (complete or partial) were seen in 54.5% (95% CI: 41.8-66.9) of the patients in the LET arm and 69.2% (95% CI: 56.6-80.1) of those in the LET + ZOL arm (*P* = 0.106). A multivariate model showed an OR of 1.72 (95% CI: 0.83-3.59) for the experimental arm.

**Conclusion:**

No increase in the clinical response rate was observed with the addition of ZOL to a neoadjuvant treatment regimen with LET. However a trend towards a better reponse in the LET + ZOL arm could be observed. This trend is consistent with previous studies that have investigated the addition of ZOL to chemotherapy, and it may support the evidence for a direct antitumor action of zoledronic acid.

## Background

Neoadjuvant therapy with either chemotherapy or antihormonal agents has increased the information available regarding the efficacy of treatment and resistance mechanisms in the primary tumor. While the evidence for the efficacy of and resistance to treatment with bisphosphonates in the neoadjuvant setting is limited the efficacy of these agents in patients with metastatic and early breast cancer has been investigated in many studies [1].

In patients with breast cancer metastatic to the bone, bisphosphonates have been shown to be effective in preventing skeletal-related events, indicating an antitumor effect [2,3]. In the adjuvant setting, three larger trials (ABSCG-12, ZO-FAST, and AZURE) have reported a prognostic benefit for at least some subgroups of breast cancer patients [4-6]. The ABCSG-12 study reported a disease-free survival (DFS) benefit in a population of premenopausal breast cancer patients when zoledronic acid was added to either anastrozole or tamoxifen, and suggested that this benefit is greatest in patients over the age of 40, who achieve a maximal estrogen blockade [5,7]. Similarly, the ZO-FAST study reported an improvement in DFS in postmenopausal breast cancer patients [6]. However, this effect was not observed in the overall population in the AZURE study [4]. Analysis of the AZURE study raised the question of which patients are able to benefit most from treatment with bisphosphonates, as specific subgroups — such as women with more than 5 years since the end of the menopause, or women with complete suppression of ovarian function — were found to experience the greatest benefit from the addition of zoledronic acid to the standard treatment [4].

Several mechanisms have been implicated that might potentially be associated with an antitumor effect of zoledronic acid; one of these is inhibition of the enzyme farnesyl diphosphate synthase, which results in altered synthesis of enzymes such Rho, Rac, and Rab that are thought to be involved in cell proliferation, cell motility, angiogenesis, and cell migration [8-15]. It has also been reported that zoledronic acid blocks the interaction between mesenchymal stem cells and breast cancer cells, which is thought to reduce the effect of mesenchymal stem cells on the progression of breast cancer [16].

On the basis of the findings from clinical studies and preclinical evidence, it was hypothesized that adding zoledronic acid to neoadjuvant treatment with letrozole might increase the response of primary breast cancer tumors. A randomized phase II trial was therefore conducted, with the primary objective being tumor response.

## Methods

The trial was conducted in accordance with the Helsinki Declaration and the International Conference on Harmonization of Technical Requirements for Registration of Pharmaceuticals for Human Use (ICH) Harmonized Tripartite Guidelines for Good Clinical Practice. The study protocol was approved by the Ethics Committee of the Medical Faculty, Friedrich-Alexander University Erlangen Nuremberg (Location of the Principal Investigator), and in addition by all relevant ethics committees at each study site, and all of the patients provided written informed consent.

### Patients

This report describes a phase II, multicenter, prospective, randomized, and controlled open-label trial. Patients had to be over the age of 18 and had to be postmenopausal as defined by age over 55; age ≤ 55 years but without a menstrual period for more than 1 year; or with luteinizing hormone and follicle-stimulating hormone levels > 40 IU/L or estradiol levels < 5 ng/dL; or had to have undergone bilateral oophorectomy before the diagnosis of breast cancer. The invasive breast cancer must have been histologically confirmed by core needle biopsy and had to have either a positive estrogen receptor (ER) and/or progesterone receptor (PgR) status, defined by core biopsy immunohistochemistry with >10% positive malignant epithelial cells. Clinical Stage had to be ≥ cT1c (Size ≥1.5 cm) without distant metastases (M0). Tumors with a size of ≥1.5 cm were allowed as target lesions, although this size is not considered as a target lesion according to RECIST criteria [17]. However tumor size assessments with mammography or breast ultrasound seem accurate enough to allow tumors of this size for evaluation [18].

Other inclusion criteria were: adequate renal function (creatinine clearance >30 mL/minute calculated using the Cockcroft-Gault equation), adequate bone-marrow function, adequate hepatic function, life expectancy of at least 12 months, and Eastern Cooperative Oncology Group (ECOG) status ≤ 2.

Exclusion criteria were: inflammatory breast cancer, prior letrozole or bisphosphonate treatment; patients with unstable angina or other uncontrolled cardiac disease; inflammatory breast cancer, evidence of distant metastases, other concurrent malignant disease, or current dental problems; and a history of diseases affecting bone metabolism.

### Treatment and tumor assessment plan

Patients were randomly assigned to one of the two neoadjuvant treatment groups — either letrozole 2.5 mg/day plus a total of seven infusions of zoledronic acid 4 mg every 4 weeks (LET + ZOL); or letrozole 2.5 mg/day only (LET) — at a ratio of 1 : 1. Surgery for the breast tumor and axillary lymph nodes was scheduled 6.5 months after the patient had received her first dose of the study treatment. Sentinel biopsies were permitted, but complete axillary dissection was mandatory if there were positive findings. If the tumor progressed during chemotherapy, the study treatment was discontinued and further treatment was at the discretion of the investigator, who was the attending physician.

The size of the tumor was assessed before the start of therapy, after 4 months of treatment, and after 6 months of treatment, in accordance with our modified response evaluation criteria (see above). All of the images were read locally. In order to avoid unblinded reader bias in this open-label study, a central review of all mammograms was additionally performed by an independent blinded radiologist in order to assess the response status of the target lesion using standardized criteria, as described below. Principally MRI and ultrasound were allowed assessment methods as per study protocol but no study site chose those imaging methods.

### End points

The primary objective of the study was explore whether the combination of letrozole (2.5 mg/day) and zoledronic acid (4 mg q4w, or dose-adjusted based on renal function) is superior to letrozole (2.5 mg/day) monotherapy in relation to the tumor response after 6 months of preoperative treatment in postmenopausal patients with primary breast cancer. “Response” was defined as a complete response (CR: complete disappearance of all target lesions) or a partial response (PR: at least a 30% decrease in the sum of the largest diameter of all target lesions), based on MRI or mammography and/or sonography in accordance with the modified RECIST criteria [17]. Tumor response evaluation was performed similar to RECIST, however lesions ≥ 1.5 cm as assessed by mammography or ultrasound were allowed as target lesions. Radiological tumor size assessment, either by ultrasound or mammography, has been shown to be very reliable in this range of tumor sizes [18-20].

Safety was primarily assessed by documentation of adverse events (AEs). The severity of AEs was classified in accordance with the National Cancer Institute (NCI) Common Terminology Criteria for Adverse Events (NCI-CTCAE version 3.0). AEs were documented during the period from first exposure to the study drug to 30 days after last exposure to it. Adverse events, whether reported by the patient, discovered during general questioning by the investigator, or detected through physical examination, laboratory tests, or other means, were recorded on the adverse event log in the study case report form and followed carefully until resolution. It was not mandatory for abnormal laboratory values or test results to be considered AEs unless they induced clinical signs and symptoms or required therapeutic interventions. Adverse events were described by duration (start and end dates, or at the final examination if continuing), severity (NCI-CTCAE grades 1–5), relationship to study drug (not suspected, suspected with letrozole, suspected with zoledronic acid, suspected with both), and action taken.

Quality of life was assessed using the FACT-B questionnaire (Functional Assessment of Cancer Therapy- Breast Cancer). The Quality of life questionnaires should be completed at each visit (baseline, month 1-6) by the patient upon arrival at the clinic and before the patient has either been interviewed by the physician or received study medication. The FACT-B was analysed in the ITT population using the ‘data as observed’ by visit as total score and separated by the sub-scores physical well-being, social/family well-being, emotional well-being, functional well-being and breast cancer subscale.

### Statistical analysis

Recruitment of a total of 850 patients was planned in order to reach a power of 80% to demonstrate the superiority of the combination therapy (LET + ZOL) in comparison with letrozole alone (LET) with regard to the response rate after 6 months. A response rate of 35% and an increase in the response by 10% with the addition of zoledronic acid was assumed for this calculation. Randomization was stratified by nodal status (N-negative vs. N-positive), tumor grading (G1 vs. G2–3) and center, and performed within strata in a 1 : 1 ratio.

A total of 168 patients were enrolled before the study was terminated due to low recruitment. Given the original assumptions, a figure of 200 patients would result in a power of only 25%, instead of the required 80%. The study was therefore grossly underpowered, and the results of the primary analysis must be regarded as exploratory rather than confirmatory.

In general, all summary statistics are presented by treatment group (LET vs. LET + ZOL). Categorical variables are summarized by absolute and relative frequencies. Continuous variables are summarized by descriptive statistics of mean, standard deviation, minimum, median, and maximum. Time-to-event data, including rates of affected patients, were assessed using Kaplan–Meier statistics.

A logistic regression model was fitted for the response, including the dichotomous factors “treatment” (LET vs. LET + ZOL), “nodal status” (N^+^ vs. N^–^), “tumor grading” (G1 vs. G2 and G3), “tumor size at baseline” (largest diameter ≤ 2 cm vs. > 2 cm) and “age” (< 65 years vs. ≥ 65 years).

The odds ratio (OR) for the combination therapy relative to the monotherapy was estimated. The difference between the treatment groups was tested using the likelihood ratio test with a two-sided significance level of 5%. *P* values are presented together with the two-sided 95% confidence intervals (CIs) for the OR.

For six patients in the LET + ZOL arm and two patients in the LET arm, a response assessment was not available after 6 months but only after 4 months. For these patients, it was assumed that the treatment response at that time would carry forward to the final assessment time point at 6 months (last observation carried forward, LOCF).

All of the statistical analyses were carried out using SAS version 8.2.

## Results

**Figure 1:**
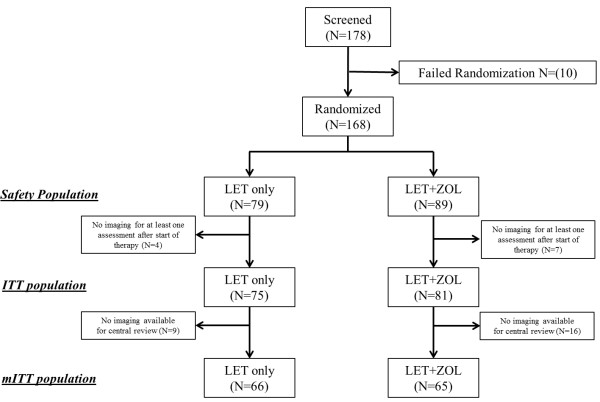
**Consolidated Standards of Reporting Trials (CONSORT) diagram.** ITT, intention to treat; mITT, modified intention to treat; LET, letrozole alone; LET + ZOL, letrozole plus zoledronic acid.

A total of 178 patients were screened for study eligibility at 27 study sites. Of these, 168 patients were randomized at 27 centers and received treatment with either letrozole monotherapy (LET; n = 79) or combination therapy with letrozole plus zoledronic acid (LET + ZOL; n = 89). This population is considered the *safety population,* in which toxicity is reported (Figure 1). Tumor measurements, assessed locally, for at least one time point after the start of treatment were available for 156 patients, and this population is considered the *intention-to-treat population* (ITT, Figure 1). Mammograms were sent to the central reader for 131 patients in the ITT group, and this population is considered as the *modified intention-to-treat population* (mITT, Figure 1). For the per-protocol (PP) analysis, an additional four patients had to be excluded (LET: 2; LET + ZOL: 2) from the mITT.

Safety is reported for all patients who started the treatment (safety population) and efficacy is reported for all patients for whom at least one mammogram was available for central assessment after the start of treatment (mITT). For the sensitivity analysis, efficacy data were analyzed for the PP and ITT populations. Patient and tumor characteristics for both treatment arms for the mITT population are summarized in Table 1 for all patients, and for the safety population in Additional file 1 Table S1.

**Table 1:** Patient and tumor characteristics for the modified intention-to-treat population

**Characteristic**	**LET (n = 66) mean ± SD or n and %**	**LET + ZOL (n = 65) mean ± SD or n and %**	**Total (n = 131) mean ± SD or n and %**
Age (years)			
Mean ± SD	70.6 ± 8.3	71.1 ± 9.1	70.8 ± 8.7
Median (range)	71.0 (54.0–89.0)	70.0 (54.0–88.0)	71.0 (54.0–89.0)
Height (cm)			
Mean ± SD	162.3 ± 6.7	162.5 ± 7.0	162.4 ± 6.8
Median (range)	163 (150–185)	163 (144–180)	163 (144–185)
Body weight (kg)			
Mean ± SD	70.7 ± 12.9	71.6 ± 16.9	71.2 ± 15.0
Median (range)	69.0 (47.0–114.0)	70.0 (41.0–150.0)	69.2 (41.0–150.0)
BMI (kg/m^2^)			
Mean ± SD	26.9 ± 4.8	27.0 ± 5.8	26.9 ± 5.3
Median (range)	26.3 (17.0–40.4)	26.2 (16.6–55.1)	26.2 (16.6–55.1)
Postmenopausal state: yes	65 (100.0)	66 (100.0)	131 (100.0)
Age group			
< 65 years	15 (22.7)	16 (24.6)	31 (23.7)
≥ 65 years	51 (77.3)	49 (75.4)	100 (76.3)
Ethnicity: Caucasian	65 (100.0)	66 (100.0)	131 (100.0)
Histological type			
Invasive ductal	44 (66.7)	48 (73.8)	92 (70.2)
Invasive lobular	12 (18.2)	8 (12.3)	20 (15.3)
Invasive ductal and lobular	2 (3.0)	3 (4.6)	5 (3.8)
Other	8 (12.1)	6 (9.2)	14 (10.7)
Grading			
G1	10 (15.2)	10 (15.4)	20 (15.3)
G2	47 (71.2)	46 (70.8)	93 (71.0)
G3	9 (13.6)	8 (12.3)	17 (13.0)
GX	0 (0.0)	1 (1.5)	1 (0.8)
T staging			
T in situ	1 (1.5)	0 (0.0)	1 (0.8)
T1	6 (9.1)	5 (7.9)	11 (8.5)
T2	48 (72.7)	40 (63.5)	88 (68.2)
T3	8 (12.1)	8 (12.7)	16 (12.4)
T4	3 (4.5)	10 (15.9)	13 (10.1)
Data lacking	0	2	2
N staging			
0	44 (66.7)	36 (55.4)	80 (61.1)
1	20 (30.3)	27 (41.5)	47 (36.0)
2	1 (1.5)	0 (0.0)	1 (0.8)
X	1 (1.5)	2 (3.1)	3 (2.3)
M staging			
0	65 (98.5)	65 (100)	130 (99.2)
X	1 (1.5)	0 (0)	1 (0.8)
Data lacking	0		
Estrogen receptor status			
Negative	2 (3.0)	2 (3.1)	4 (3.1)
Positive	64 (97.0)	63 (96.9)	127 (96.9)
Progesterone receptor status			
Negative	7 (10.6)	6 (9.2)	13 (9.9)
Positive	59 (89.4)	59 (90.8)	118 (90.1)
ECOG performance status			
0	42 (63.6)	38 (58.5)	80 (61.1)
1	20 (30.3)	24 (36.9)	44 (33.6)
2	4 (6.1)	3 (4.6)	7 (5.3)
3	0 (0)	0 (0.0)	0 (0)

BMI, body mass index; ECOG, Eastern Cooperative Oncology Group; LET, letrozole alone; LET + ZOL, letrozole plus zoledronic acid; SD, standard deviation.

The patients’ average age was 70.8 years. Most of the patients (87.7%) had a tumor stage above cT1. As the enrolled patients represent an older population, most (86.3%) had a concomitant medical condition — mainly vascular disorders (56.5%), metabolic and nutritional disorders (32.7%), and musculoskeletal disorders (24.4%).

### Efficacy

**Figure 2:**
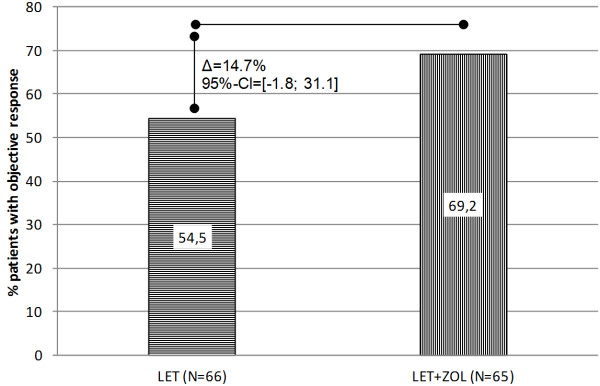
Primary efficacy analysis: Response Evaluation Criteria in Solid Tumors (RECIST) tumor response rates (complete response + partial response) at month 6, based on the central review (modified intention to treat, last observation carried forward).

The primary efficacy variable was tumor response (CR + PR) after 6 months of neoadjuvant treatment. In the LET-only arm, there were no clinical complete responses and 36 patients (54.5%) had a partial response. None of the patients had progressive disease. In the LET + ZOL arm, there were two patients (3.1%) with a complete response and 43 patients (66.2%) with a partial response (Figure 2). One patient (1.5%) was reported to have progression. With regard to the primary end point, the response rate in the LET-only arm was therefore 54.5% (95% CI, 41.8 to 66.9), in comparison with 69.2% (95% CI, 56.6 to 80.1) in the LET + ZOL arm. The *P* value for the difference was 0.106. However, this primary analysis was underpowered due to the insufficient study recruitment. The mean target lesion size (clinically assessed, longest diameter) decreased by 1.12 cm (±0.92) from 3.23 cm (±1.19) to 2.12 cm (±1.04) in the LET only arm and decreased by 1.37 cm (±0.96) from 3.45 cm (±2.54) cm to 2.08 cm (±2.27) in the LET + ZOL arm.

With regard to histopathological assessment one patient in the LET + ZOL arm had a regression to a carcinoma in situ with no invasive tumor components, and no pathological complete response was observed. Pathological tumor sizes and pathological ypT classification are shown in Table 2 for the mITT population and Additional file 2 Table S2 for the safety population.

**Table 2:** Tumor characteristics at the time of surgery for the modified intention to treat population

**Characteristic**	**LET (n = 66) mean ± SD or n and %**	**LET + ZOL (n = 65) mean ± SD or n and %**	**Total (n = 131) mean ± SD or n and %**
Surgery performed			
No	7 (10.6)	3 (4.6)	10 (7.6)
Yes	59 (89.4)	62 (95.4)	121 (92.4)
unknown	0	0	0
pCR			
No	59 (100)	62 (100)	121 (100)
Yes	0 (0.0)	0 (0.0)	0 (0.0)
Unknown	7	3	10
ypT			
0	0 (0)	0 (0)	0 (0)
is	0 (0)	1 (1.6)	1 (0.8)
1	23 (39.0)	23 (37.1)	46 (38.0)
2	29 (49.2)	28 (45.2)	57 (47.1)
3	6 (10.2)	5 (8.1)	11 (9.1)
4	1 (1.7)	5 (8.1)	6 (5.0)
Unknown	7	3	10
Mean patholocigal tumor size in cm	2.9 (±2.0)	2.7 (±1.7)	2.8 (±1.8)

In the logistic regression model, none of the covariates mentioned above (Table 3) was associated with a response. The analysis showed a trend in favor of the LET + ZOL treatment (OR = 1.72; 95% CI, 0.827 to 3.586; *P* = 0.147).

**Table 3:** Logistic regression model for predicting tumor response

	**n**	**Logistic regression OR [95% CI] (*P *** **value)**
Nodal status		
N0	51	1 (ref)
N1	80	0.962 [0.446; 2.078] (0.922)
Grading		
G2–3	110	1 (ref)
G1	20	1.203 [0.433; 3.339] (0.723)
Baseline tumor size		
> 2 cm	16	1 (ref)
≤ 2 cm	114	0.742 [0.242; 2.274] (0.601)
Age		
≥ 65 years	100	1 (ref)
< 65 years	31	1.189 [0.483; 2.924] (0.707)
Treatment		
LET	65	1 (ref)
LET + ZOL	66	1.722 [0.827; 3.586] (0.147)

The per-protocol analysis, using central imaging assessments, showed a clinical response in 35 out of 64 patients (54.7%; 95% CI, 41.7 to 67.2) in the LET-only arm and in 43 out of 63 patients (68.3%; 95% CI, 55.3 to 79.4) in the LET + ZOL arm (*P* = 0.145). Using the tumor assessment at the local study sites (ITT population), a response was seen in 26 out of 75 patients (34.7%; 95% CI, 24.0 to 46.5) in the LET arm and in 37 out of 81 patients (45.7%; 95% CI, 34.6 to 57.1) in the LET + ZOL arm (*P* = 0.192).

### Effect on surgery

A total of 12 patients in the ITT population did not undergo surgery- Documented primary reasons were "wish of the patient" (10 patients), "other" (0 patients), and "inoperable" (2 patients).

A review of the 42 ITT patients who were scheduled at Baseline to undergo "mastectomy" shows that 13 patients (31.0%) actually underwent radical mastectomy, while 6 patients (14.3%) were not operated, 4 patients (9.5%) were treated with modified radical mastectomy, 18 patients (42.9%) had a lumpectomy/quadrantectomy, and 1 patient (2.4%) was treated with "other" methods. The vice versa rates of patients switched from planned lumpectomy/quadrantectomy to radical and modified radical mastectomy were 7.9% (n = 9) and 6.1% (n = 7). Neither treatment arm or other factors such as nodal status, age, grading or tumor size at baseline were associated with type of performed surgery.

### Compliance and quality of life

A total of 27 patients (LET: 12; LET + ZOL: 15) discontinued the study prematurely. In the LET arm, this was due to an unsatisfactory treatment effect (n = 4), an adverse event (n = 1), withdrawal of consent by the patient (n = 6), and abnormal laboratory values (n = 1). In the LET + ZOL arm, it was due to an unsatisfactory treatment effect (n = 4), adverse events (n = 6), withdrawal of consent by the patient (n = 1), abnormal laboratory values (n = 1), protocol violations (n = 2), and administrative problems (n = 1).

The mean duration of letrozole administration in the ITT population (n = 168) was 174.2 ± 39.5 days. Calculating 1 month as 28 days, the median duration of treatment was exactly 6.5 months, as planned in the study protocol. Of the patients receiving the combination therapy, 70.8% received all seven (six or more) scheduled infusions. The mean duration of exposure was 177.2 ± 43.4 days.

The mean baseline total score in the Functional Assessment of Cancer Therapy-Breast (FACT-B) in the ITT population was 111.4 ± 18.5 score points (median: 115.0, range: 51.4–141.0), with no relevant differences between the treatment groups. This baseline score remained stable over time up to month 6 (with a change by –1.8 ± 13.4 score points). Similarly, the analysis of subscores also did not show any relevant change during the study period at any assessment time point.

### Safety

A total of 64 patients (81.%) reported Grade 1 or 2 adverse events (AE) in the LET only arm and 78 (87.6%) in the LET + ZOL arm. With regard to grade 3 or 4 AEs, 7 patients (8.9%) were reported in the LET only arm and 17 patients (19.1%) in the LET + ZOL arm.

Most frequent side effects were musculoskeletal disorders, hot flushes, skin and gastrointestinal disorders. An overview of AEs is given in Table 4.

**Table 4:** Toxicities according to NCI-CTCAE Version 3.0 in the safety population

		**LET (n = 79) n (%)**		**LET + ZOL (n = 89) n (%)**
	Grade 1 and 2	Grade 3 and 4	Grade 1 and 2	Grade 3 and 4
Musculoskeletal and connective tissue disorders	29 (36.7)	2 (2.5)	39 (43.8)	4 (4.5)
Hot flush	25 (31.6)	2 (2.5)	19 (21.3)	0 (0.0)
Skin and subcutaneous tissue disorders	18 (22.8)	0 (0.0)	18 (20.2)	1 (1.1)
Gastrointestinal disorders	16 (20.3)	2 (2.5)	13 (14.6)	2 (2.2)
Infections and infestations	14 (17.7)	1 (1.3)	16 (18.0)	0 (0.0)
Psychiatric disorders	13 (16.5)	2 (2.5)	14 (15.7)	1 (1.1)
Fatigue	12 (15.2)	1 (1.3)	19 (21.3)	1 (1.1)
Nervous system disorders	12 (15.2)	1 (1.3)	18 (20.2)	3 (3.4)
Vertigo and Nausea	11 (13.9)	1 (1.3)	16 (18.0)	2 (2.2)
Cardiac disorders	4 (5.1)	1 (1.3)	4 (4.5)	0 (0.0)
Metabolism and nutrition disorders	4 (5.1)	0 (0.0)	10 (11.2)	0 (0.0)
Respiratory, thoracic and mediastinal disorders	3 (3.8)	1 (1.3)	4 (4.5)	2 (2.2)
Fractures	2 (2.6)	2 (2.5)	0 (0.0)	2 (2.2)
Vomiting	1 (1.3)	0 (0.0)	4 (4.5)	0 (0.0)
Pyrexia	1 (1.3)	0 (0.0)	5 (5.6)	0 (0.0)
Renal and urinary disorders	1 (1.1)	0 (0.0)	5 (6.3)	0 (0.0)
Blood and lymphatic system disorders	0 (0.0)	1 (1.3)	2 (2.2)	1 (1.1)
Hepatobiliary disorders	0 (0.0)	0 (0.0)	1 (1.1)	0 (0.0)

## Discussion

In this prematurely terminated, randomized phase II study in postmenopausal women with hormone receptor–positive primary breast cancer, a positive trend in the clinical tumor response was observed with the addition of zoledronic acid to neoadjuvant treatment with letrozole. This is to the best of our knowledge the first study in neoadjuvant breast cancer that has examined the effect of zoledronic acid in addition to endocrine treatment. The study was terminated due to insufficient recruitment. The treatment was well tolerated and did not affect the patients’ quality of life.

The response rate in the LET-only arm was within the range of response rates reported in other neoadjuvant studies that have examined the response to neoadjuvant therapy with aromatase inhibitors. The response rates in these studies have varied from 37% to 85% [21-26]. This wide range reflects a problem that is associated with conducting neoadjuvant anti-endocrine studies. The study population is unlikely to achieve a pathological complete response (pCR), with both chemotherapy and anti-endocrine therapy. In neoadjuvant anti-endocrine studies, the clinical response rate (cRR) is therefore used as an end point [27,28], leading to reduced comparability between studies and observers. This can be seen in the present study as well, as there was a difference between the central assessment and the local one.

Although the difference between the response rates in the two treatment arms in the present study was not significant, the direction of the observed effect is the same as in two previously published studies. In the Adjuvant Zoledronic Acid to Reduce Recurrence (AZURE) study, the subgroup of neoadjuvantly treated breast cancer patients was analyzed [29]. In this retrospective and exploratory subgroup analysis of 205 patients, the tumor size was compared between patients who received chemotherapy (n = 103) and patients who received chemotherapy and zoledronic acid (n = 102). The tumor size at the time of surgery was smaller in the zoledronic acid plus chemotherapy group than in the chemotherapy-only group. The residual invasive tumor size (RITS) in the chemotherapy group was found to be 27.4 mm and the RITS in the chemotherapy plus zoledronic acid group was found to be 15.5 mm. This difference was statistically significant (*P* = 0.006). There was a nonsignificant difference with regard to the pCR, with 6.9% in the chemotherapy group and 11.7% in the chemotherapy plus zoledronic acid group (*P* = 0.146). The pCR rates are rather low, but this is representative for this population of patients. Similarly, a retrospective cohort analysis in breast cancer patients documented in the M.D. Anderson Cancer Center database identified 39 patients who were treated with chemotherapy and neoadjuvant bisphosphonates. These patients had a pCR in 25.4% of cases, in comparison with only 16% in the group who did not receive a bisphosphonate (*P* = 0.11) [30]. This difference resulted in an OR of 2.18 (95% CI, 0.9 to 5.24; *P* = 0.08) in favor of the group treated with zoledronic acid. Thus, this study suggests a possible direct antitumor effect of zoledronic acid as well. In another neoadjuvant trial (NEOZOTAC Trial) ZOL was added to a taxane, anthracycline and cyclophosphamide based chemotherapy. No significant change in pCR rate could be observed, but a nominal positive effect could be seen in a subgroup of postmenopausal patients [31]. Comparable results could be shown in a Japanese study comparing chemotherapy (FEC100 q3w x4 followed by weekly paclitaxel for 12 cycles) with or without ZOL. No significant change in pCR could be reached, but a trend could be observed in postmenopausal patients with triple-negative breast cancer [32].

The present study is the first to investigate the addition of a bisphosphonate to a neoadjuvant endocrine therapy, showing similar results. As in the other two studies discussed above, the sample size may have been too low to detect a statistically significant difference.

The group of patients included was older, with an average age of 70 years. The group treated may represent a subset of patients who are thought to be more susceptible to bisphosphonate treatment than other subgroups. In addition to the ABSCG-12 trial and the ZO-FAST trial, which reported a potential anticancer effect of zoledronic acid [5,6], the AZURE study has also raised the question of which patients benefit most from the treatment [4]. The AZURE study included premenopausal and postmenopausal patients who received either chemotherapy, antihormonal therapy, or both, and they were randomly assigned to treatment with zoledronic acid. There was no difference in the disease-free survival in the overall study population (HR 0.98; *P* = 0.79) [4]. However, in patients who were described as having a low-estrogen environment — defined as either being aged 60 or older or being at least 5 years past the menopause — a significant improvement in the disease-free survival was observed [4]. The population included in the present study fully meets all the criteria for this subgroup. In addition, the patients in the present study received antihormonal treatment, which may suppress any estrogen activity in the tumor even more. It should be borne in mind that the results should be seen in this context and that the already weak findings should be interpreted with caution and only for this subgroup of patients.

There are some obvious limitations to the study, one being its premature termination due to insufficient recruitment. The study was designed with an assumed 35% response rate to monotherapy with letrozole; this expectation in the sample size estimation was clearly exceeded (54.5%), and the stipulated clinically relevant difference of 10% in favor of the combination treatment was met, with a difference in the response rate of 14.7%. However, statistical significance was not reached, most likely because of the low sample size resulting from the insufficient recruitment rate. In addition, as in other neoadjuvant anti-endocrine studies, the assessment of the tumor response was not standardized. The response rates in the local assessment were 34.7% in the LET-only arm and 45.7% in the LET + ZOL arm, in comparison with 54.5% and 69.2%, respectively, in the central assessment. Although the effect observed was in the same direction, the response rate and response rate difference were smaller in the local assessment. The results thus reflect the difficulty of assessing the tumor response in neoadjuvant antihormone therapy studies. However, sensitivity analyses showed similar effects for all analyses. Due to rare complete pathological responses after neoadjuvant anti-endocrine therapy the primary endpoint had to be a clinical one. In our study we did not see any pCR. Although central assessment was considered to reduce interobserver variability, a discordance between the central and the local assessment has to be mentioned. There were 22 patients for which the local assessment of a stable disease was changed to partial response and there were 8 patients for whom the local assessment of partial response was changed to stable disease. Other discordances, that would have an effect on the primary study aim “response” were not made. In addition to the radiological assessment one could consider the comparison of pathological tumor sizes. No statistical significant difference was observed concerning this parameter.

## Conclusions

In summary, this study was unable to show the superiority of neoadjuvant treatment with letrozole and zoledronic acid over letrozole-only therapy in relation to the clinical response rate. Although the study suggests a positive effect, the number of patients recruited in this prematurely terminated study was too small for the effect to be shown to be significant. The early termination of the study is explained by the difficulty to recruit patients for neoadjuvant anti-endocrine studies, particularly since novel targeted therapies are evolving that address the problem of treatment resistance in this group of patients with a generally low response rate. However, the study results are suggestive of a direct antitumor effect of zoledronic acid.

## Competing Interests

We are grateful to Novartis Germany for financial support for the trial and for supplying the medication.

The authors declare the following conflict of interests: This study was sponsored and funded by Novartis. Peter A. Fasching has received honoraria from Novartis and conducts Novartis-funded research. Christopher Wolf received honoraria from Novartis. Volker Hanf has reveiced honoraria from Novartis. Stefan Paepke has received Honoraria from Novartis. Thorsten Kühn has received honoraria from Novartis and is a member of advisory boards of Novartis. Rolf Kreienberg is a member of advisory boards of Novartis. Michael P. Lux has received honoraria from Novartis and conducts Novartis-funded research. Matthias W. Beckmann has conducted Novartis funded research and is advisory board member for Novartis and Pfizer. Mathias Muth, Monika Baier, and Birgit Wackwitz are employees of Novartis Pharma GmbH, Nuremberg, Germany. All other authors declare that they have competing interest.

## Authors’ contributions

PAF drafted the manuscript, approved the final manuscript, designed the study, acquired data and recruited a substantial part of the patients. SMJ approved the final manuscript, acquired central imaging data. MH approved the final manuscript, acquired data and recruited a substantial part of the patients. SK approved the final manuscript, acquired data and recruited a substantial part of the patients. MS approved the final manuscript, acquired data and recruited a substantial part of the patients. MW approved the final manuscript, acquired data and recruited a substantial part of the patients. VH approved the final manuscript, acquired data and recruited a substantial part of the patients. DG approved the final manuscript, acquired data and recruited a substantial part of the patients. JK approved the final manuscript, acquired data and recruited a substantial part of the patients. ES approved the final manuscript, acquired data and recruited a substantial part of the patients. RK approved the final manuscript, acquired data and recruited a substantial part of the patients. TM approved the final manuscript, acquired data and recruited a substantial part of the patients. TK approved the final manuscript, acquired data and recruited a substantial part of the patients. CW approved the final manuscript, acquired data and recruited a substantial part of the patients. SK approved the final manuscript, acquired data and recruited a substantial part of the patients. SP approved the final manuscript, acquired data and recruited a substantial part of the patients. MB approved the final manuscript, acquired data and recruited a substantial part of the patients. MM designed the study, drafted the manuscript and approved the final manuscript. MB performed the statistical analysis. BW designed the study, drafted the manuscript and approved the final manuscript. RSW performed the central review of the mammographies and approved the final manuscript. MWB drafted the manuscript, approved the final manuscript, designed the study, acquired data and recruited a substantial part of the patients. MPL drafted the manuscript, approved the final manuscript, designed the study, acquired data and recruited a substantial part of the patients. All authors read and approved the final manuscript.

## Pre-publication history

The pre-publication history for this paper can be accessed here:

http://www.biomedcentral.com/1471-2407/14/66/prepub

## Supplementary Material

Additional file 1: Table S1 Patient and tumor characteristics in the safety population. Click here for file

Additional file 2: Table S2 Tumor characteristics at the time of surgery for the safety population. Click here for file
