# Supplementary material for: FemZone trial: a randomized phase II trial comparing neoadjuvant letrozole and zoledronic acid with letrozole in primary breast cancer patients
Source: BMC Cancer. 2014 Feb 5;14:66. doi: 10.1186/1471-2407-14-66 (PMC3937056; doi:10.1186/1471-2407-14-66)
Supplement: Additional file 1: Table S1 — Patient and tumor characteristics in the safety population. [file 1471-2407-14-66-S1.doc]

**Additional file 1: Table S1**. Patient and tumor characteristics in the safety population

| Characteristic | LET (n = 79) Mean ± SD or n and % | LET+ZOL (n = 89) Mean ± SD or n and % | Total (n = 168) Mean ± SD or n and % |
| --- | --- | --- | --- |
| Age (years) |  |  |  |
| Mean ± SD | 70.5 ± 8.4 | 71.3 ± 9.2 | 70.9 ± 8.8 |
| Median (range) | 71.0 (54.0–89.0) | 70.0 (54.0–89.0) | 71.0 (54.0–89.0) |
| Height (cm) |  |  |  |
| Mean ± SD | 161.7 ± 7.4 | 162.1 ± 7.0 | 161.9 ± 7.2 |
| Median (range) | 163.0 (135.0–185.0) | 162.0 (144.0–180.0) | 163.0 (135.0–185.0) |
| Body weight (kg) |  |  |  |
| Mean ± SD | 70.3 ± 12.6 | 69.7 ± 15.7 | 70.0 ± 14.3 |
| Median (range) | 69.0 (47.0–114.0) | 67.0 (41.0–150.0) | 68.2 (41.0–150.0) |
| BMI (kg/m^2^) |  |  |  |
| Mean ± SD | 27.0 ± 5.1 | 26.5 ± 5.4 | 26.7 ± 5.3 |
| Median (range) | 26.0 (17.0–40.4 | 25.9 (16.6–55.1) | 25.9 (16.6–55.1) |
| Postmenopausal state: yes | 79 (100.0) | 89 (100.0) | 168 (100.0) |
| Age group |  |  |  |
| < 65 years | 19 (24.1) | 20 (22.5) | 39 (23.2) |
| ≥ 65 years | 60 (75.9) | 69 (77.5) | 129 (76.8) |
| Ethnicity: Caucasian | 79 (100.0) | 89 (100.0) | 168 (100.0) |
| Histological type |  |  |  |
| Invasive ductal | 53 (67.1) | 63 (70.8) | 116 (69.0) |
| Invasive lobular | 15 (19.0) | 16 (18.0) | 31 (18.5) |
| Invasive ductal and lobular | 2 (2.5) | 3 (3.4) | 5 (3.0) |
| Other | 9 (11.4) | 7 (7.9) | 16 (9.5) |
| Grading |  |  |  |
| G1 | 11 (14.1) | 14 (15.7) | 25 (15.0) |
| G2 | 58 (74.4) | 66 (74.2) | 124 (74.3) |
| G3 | 9 (11.5) | 8 (9.0) | 17 (10.2) |
| GX | 0 (0.0) | 1 (1.1) | 1 (0.6) |
| Data lacking | 1 | 0 | 1 |
| T staging |  |  |  |
| T in situ | 1 (1.3) | 0 (0.0) | 1 (0.6) |
| T1 | 6 (7.7) | 5 (5.9) | 11 (6.7) |
| T2 | 58 (74.4) | 56 (65.9) | 114 (69.9) |
| T3 | 9 (11.5) | 11 (12.9) | 20 (12.2) |
| T4 | 4 (5.1) | 13 (15.3) | 17 (10.4) |
| Data lacking | 1 | 4 | 5 |
| N staging |  |  |  |
| 0 | 56 (70.9) | 53 (60.2) | 109 (65.3) |
| 1 | 21 (26.6) | 31 (35.2) | 52 (31.1) |
| 2 | 1 (1.3) | 0 (0.0) | 1 (0.6) |
| X | 1 (1.3) | 2 (2.3) | 3 (1.8) |
| Data lacking | 0 | 1 | 1 |
| M staging |  |  |  |
| 0 | 78 (98.7) | 86 (98.9) | 164 (98.8) |
| x | 1 (1.3) | 1 (1.1) | 2 (1.2) |
| Data lacking | 0 | 2 | 2 |
| Estrogen receptor status |  |  |  |
| Negative | 2 (2.5) | 2 (2.2) | 4 (2.4) |
| Positive | 77 (97.5) | 87 (97.8) | 164 (97.6) |
| Progesterone receptor status |  |  |  |
| Negative | 8 (10.1) | 11 (12.4) | 19 (11.3) |
| Positive | 71 (89.9) | 78 (87.6) | 149 (88.7) |
| ECOG performance status |  |  |  |
| 0 | 50 (63.3) | 52 (58.4) | 102 (60.7) |
| 1 | 24 (30.4) | 31 (34.8) | 55 (32.7) |
| 2 | 4 (5.1) | 6 (6.7) | 10 (6.0) |
| 3 | 1 (1.3) | 0 (0.0) | 1 (0.6) |

BMI, body mass index; ECOG, Eastern Cooperative Oncology Group; LET, letrozole alone; LET+ZOL, letrozole plus zoledronic acid; SD, standard deviation.
