# Supplementary material for: FemZone trial: a randomized phase II trial comparing neoadjuvant letrozole and zoledronic acid with letrozole in primary breast cancer patients
Source: BMC Cancer. 2014 Feb 5;14:66. doi: 10.1186/1471-2407-14-66 (PMC3937056; doi:10.1186/1471-2407-14-66)
Supplement: Additional file 2: Table S2 — Tumor characteristics at the time of surgery for the safety population. [file 1471-2407-14-66-S2.doc]

**Additional file 2: Table S2**: Tumor characteristics at the time of surgery for the safety population

| Characteristic | LET (n = 79) Mean ± SD or n and % | LET+ZOL (n = 89) Mean ± SD or n and % | Total (n = 168) Mean ± SD or n and % |
| --- | --- | --- | --- |
|  |  |  |  |
| Surgery performed |  |  |  |
| No | 10 (12.7) | 9 (10.1) | 19 (12.0) |
| Yes | 69 (87.3) | 80 (89.9) | 149 (94.3) |
| unknown | 0 | 0 | 0 |
|  |  |  |  |
| pCR |  |  |  |
| No | 67 (100) | 80 (100) | 147 (100) |
| Yes | 0 (0) | 0 (0) | 0 (0) |
| unknown | 12 | 9 | 21 |
|  |  |  |  |
| ypT |  |  |  |
| 0 | 0 (0) | 0 (0) | 0 (0) |
| Is | 0 (0) | 1 (1.3) | 1 (0.7) |
| 1 | 27 (39.7) | 31 (38.8) | 58 (39.2) |
| 2 | 34 (50.0) | 34 (42.5) | 68 (45.9) |
| 3 | 6 (8.8) | 9 (11.3) | 15 (10.1) |
| 4 | 1 (1.5) | 5 (6.3) | 6 (4.1) |
| unknown | 12 | 9 | 21 |
|  |  |  |  |
| Mean patholocigal tumor sizein cm | 2.7 (±1.9) | 2.9 (±2.0) | 2.8 (±1.9) |
